# Early breast cancer in young *BRCA* carriers: from diagnosis to treatment and survivorship

**DOI:** 10.1177/17588359251368733

**Published:** 2025-09-17

**Authors:** Roberto Borea, Luca Arecco, Simone Nardin, Edoardo Chiappe, Chiara Lanzavecchia, Mónica Fragío Gil, Arianna Meacci, Giang Pham Hoang, Luiza Nardin Weis, Luciana de Moura Leite, Valentina Eva Klocker, Pedro Freire, Nicole Brunetti, Lucia Trevisan, Giulia Buzzatti, Stefano Spinaci, Matteo Lambertini

**Affiliations:** Department of Medical Oncology, Clinica di Oncologia Medica, IRCCS Ospedale Policlinico San Martino, Genova, Italy; Department of Internal Medicine and Medical Specialties (DIMI), School of Medicine, University of Genova, Genova, Italy; Department of Internal Medicine and Medical Specialties (DIMI), School of Medicine, University of Genova, Genova, Italy; Université libre de Bruxelles (ULB), Hôpital Universitaire de Bruxelles (H.U.B), Institut Jules Bordet, Academic Trials Promoting Team, Bruxelles, Belgium; Department of Medical Oncology, Clinica di Oncologia Medica, IRCCS Ospedale Policlinico San Martino, Genova, Italy; Department of Internal Medicine and Medical Specialties (DIMI), School of Medicine, University of Genova, Genova, Italy; Department of Medical Oncology, Clinica di Oncologia Medica, IRCCS Ospedale Policlinico San Martino, Genova, Italy; Department of Internal Medicine and Medical Specialties (DIMI), School of Medicine, University of Genova, Genova, Italy; Department of Medical Oncology, Clinica di Oncologia Medica, IRCCS Ospedale Policlinico San Martino, Genova, Italy; Department of Internal Medicine and Medical Specialties (DIMI), School of Medicine, University of Genova, Genova, Italy; Department of Medical Oncology, Clinica di Oncologia Medica, IRCCS Ospedale Policlinico San Martino, Genova, Italy; Department of Medical Oncology, Hospital Universitario Parc Tauli, Sabadell, Spain; Department of Medical Oncology, Clinica di Oncologia Medica, IRCCS Ospedale Policlinico San Martino, Genova, Italy; Department of Translational and Precision Medicine, Tor Vergata University of Rome, Rome, Italy; Department of Oncology, Vinmec International General Hospital, Hanoi, Vietnam; DASA Oncology, Brasilia, Brazil; Department of Medical Oncology, A.C. Camargo Cancer Center, São Paulo, Brazil; Department of Internal Medicine, Division of Oncology, Medical University of Graz, Graz, Austria; Department of Medical Oncology, Oncologia D’Or, Recife, Brazil; Department of Radiology, IRCCS Ospedale Policlinico San Martino, Genoa, Italy; Department of Experimental Medicine, University of Genoa, Genoa, Italy; Department of Medical Oncology, Hereditary Cancer Unit, IRCCS Ospedale Policlinico San Martino, Genoa, Italy; Department of Medical Oncology, Hereditary Cancer Unit, IRCCS Ospedale Policlinico San Martino, Genoa, Italy; Chirurgia Senologica, Ospedale Villa Scassi, ASL3, Genova, Italy; Department of Medical Oncology, Clinica di Oncologia Medica, IRCCS Ospedale Policlinico San Martino, Largo Rosanna Benzi 10, Genova 16132, Italy; Department of Internal Medicine and Medical Specialties (DIMI), School of Medicine, University of Genova, Genova, Italy

**Keywords:** BRCA, breast cancer, genetic test, treatment, young

## Abstract

Breast cancer is the most frequent malignancy among young women, with unique challenges particularly for carriers of *BRCA* pathogenic or likely pathogenic variants (PVs). Among them, the indication for intensive surveillance and risk-reducing surgeries is critical. In addition, special considerations on systemic treatment should be considered, including the use of targeted treatments like poly (adenosine diphosphate (ADP)-ribose) polymerase inhibitors. Moreover, the impact of anticancer treatments and risk-reducing surgeries on their ovarian reserve, pregnancy wish, and breastfeeding is a crucial aspect to be considered. Proper management of long-term toxicities, such as bone and cardiovascular health, as well as menopause-related symptoms, requires proper multidisciplinary care to optimize quality of life. This review examines the biological, clinical, therapeutic, and survivorship implications of breast cancer in young *BRCA* carriers, emphasizing differences between carriers of PVs in the *BRCA1* and *BRCA2* genes. Personalized strategies integrating genetic counseling, tailored surveillance and survivorship programs, as well as innovative therapies, are essential for improving prognosis and well-being in these young patients. Multidisciplinary care and further academic research efforts are critical to improve the management of breast cancer in young *BRCA* carriers.

## Introduction

**Figure 1.**
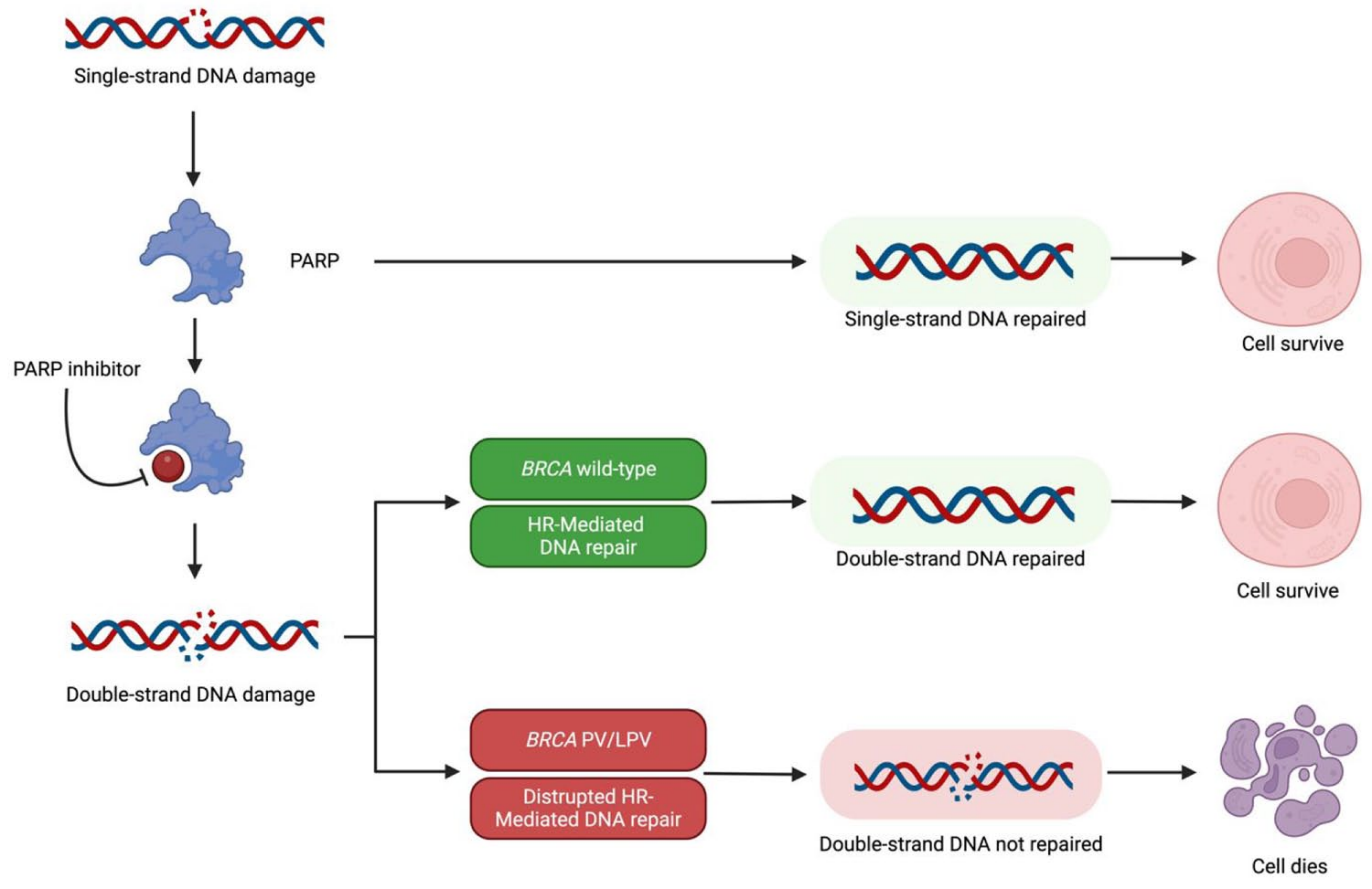
DNA repair mechanisms. Source: Created in BioRender.^6^ HR, homologous recombination; LPV, likely pathogenic variant; PARP, poly (ADP-ribose) polymerase; PV, pathogenic variant.

Breast cancer is the most frequent malignancy among women, with different incidence depending on genetic and environmental factors.^1^ Among inherited causes, pathogenetic or likely pathogenic variants (PVs) in the *BRCA1* or *BRCA2* genes are particularly relevant, contributing to a significantly increased risk of developing breast cancer and other neoplasms.^2^ BRCA proteins function as tumor suppressors and play a crucial role in repairing double-stranded DNA breaks (DSBs) through homologous recombination (HR) mechanisms.^3^ These genes are essential to maintain genomic stability and are central to the HR-mediated repair of DSBs. BRCA proteins support the HR process by facilitating the formation of the RAD51-BRCA2-DSS1 complex, which is crucial for accurate DNA repair.^4^ DSBs can arise during DNA replication due to metabolic stress. When such damage occurs, the DNA damage response (DDR) is activated and mediated by key kinases such as ataxia-telangiectasia-mutated (ATM), ataxia telangiectasia-mutated and Rad3-related (ATR), and checkpoint kinases (CHK1/2).^5^ This activation triggers cell cycle arrest and initiates DNA repair mechanisms to restore genomic integrity (Figure 1).

Although *BRCA* PVs are relatively rare in the general population, their prevalence is higher among young women affected by breast cancer.^7,8^ Between 10% to 15% of young women (defined as ⩽40 years) with newly diagnosed breast cancer are expected to carry a germline *BRCA* PV.^7^ The introduction of large-scale genetic testing has led to the identification of a growing number of individuals at increased cancer risk, guiding preventive strategies, including intensive surveillance and risk-reducing surgeries.^9^ At the same time, the peculiarities of breast cancer in *BRCA* carriers, such as increased sensitivity to DNA-damaging drugs and poly (ADP-ribose) polymerase inhibitors (PARPi), emphasize the need for tailored therapeutic approaches.^10^ Moreover, the impact of oncological treatments and risk-reducing surgeries on ovarian reserve, pregnancy wish, and breastfeeding is a critical issue to be considered.^11^ Proper management of long-term toxicities, such as bone and cardiovascular health, as well as menopause-related symptoms, requires proper multidisciplinary care to optimize quality of life.^12^

The aim of this manuscript is to review the key features of breast cancer management in young *BRCA* carriers, focusing on its biological, clinical, therapeutic, and survivorship implications. Particular attention is given to the different clinical behaviors of breast cancer according to the specific *BRCA* gene, thus differentiating between *BRCA1* and *BRCA2* carriers.

## Germline genetic testing and implications on surveillance and preventive strategies

### Genetic testing

Sequencing the *BRCA* genes in the 1990s marked a significant breakthrough in understanding the genetic basis of breast cancer, with subsequent important implications for its clinical management.^13^ Since then, indications for genetic testing and guidelines about the management of individuals carrying PVs in the *BRCA* genes have constantly evolved.^14^ Nowadays, 3% of all patients with breast cancer are expected to carry a PV in the *BRCA* genes.^15,16^ *BRCA1* carriers are estimated to have a 60%–72% chance of developing breast cancer, and, for *BRCA2* carriers, the risk is approximately 55%–69%.^17,18^ Identifying a PV in the *BRCA* genes offers major implications for these individuals and their family members, opening the door for intensive surveillance programs that include breast magnetic resonance imaging (MRI) and preventive measures such as risk-reducing bilateral mastectomy (RRBM) and risk-reducing salpingo-oophorectomy (RRSO).^19^ Moreover, individuals undergoing genetic testing should be informed about the reasons for the test, the potential outcomes, and its possible implications for other family members.^20^

According to international guidelines, there is a noticeable trend toward broader access to genetic testing compared to the past. The recently updated guidelines by the American Society of Clinical Oncology (ASCO) recommend performing the test in all newly diagnosed patients with breast cancer younger than or equal to 65 years of age at diagnosis, and in selected patients over 65 years of age based on personal and/or family history, or for those candidates for PARPi.^14^ In addition, the test is indicated in patients who develop a second breast cancer in the ipsilateral or contralateral breast.^14^ Table 1 shows the main criteria for referring patients with breast cancer for a genetic evaluation according to the American Society of Clinical Oncology,^14^ the National Comprehensive Cancer Network (NCCN) guidelines,^21^ and the European Society for Medical Oncology (ESMO) guidelines.^19^

**Table 1.** Criteria for genetic testing according to the main international guidelines.

Patient characteristics	ASCO	ESMO	NCCN
Age	⩽65 years for all patients diagnosed with breast cancer (any stage)	*National criteria	⩽65 years for all patients with breast cancer (any stage)
Sex	All male patients	All male patients	All male patients
Subtype	All cases of TNBC, regardless of age	*National criteria	TNBC diagnosed before the age of 60
Treatment	Candidates for PARPi therapy for early-stage or metastatic disease	Candidates for PARPi therapy for early-stage or metastatic disease	Candidates for PARPi therapy for early-stage or metastatic disease
Second BC	Second breast neoplasm in the ipsilateral or contralateral breast	Not specified	Multiple breast tumors (synchronous or metachronous)
Family history	Any family history suspected of *BRCA* variants, nonspecified	Three or more breast and/or ovarian cancer cases, at least one <50 years	⩾1 close blood relative with ANY: ▪ Breast cancer at age ⩽50 years ▪ Male breast cancer ▪ Ovarian cancer ▪ Pancreatic cancer ▪ Prostate cancer with metastatic, or high- or very-high-risk group
		Two breast cancer cases <40 years	⩾3 diagnoses of breast and/or prostate cancer (any grade) on the same side of the family, including the patient with breast cancer
Ethnicity	Ashkenazi Jewish ancestry or members of a population with an increased prevalence of founder mutations	Ashkenazi Jewish with breast cancer of <60 years; young-onset bilateral breast cancer, and breast and ovarian cancer in the same patient	Ashkenazi Jewish ancestry or members of a population with an increased prevalence of founder mutations
Other criteria	—	—	Multiple primary breast cancers (synchronous or metachronous)
			Individuals with any blood relative with a known PV in a cancer susceptibility gene
Multigene panel	Consider testing for high-penetrance genes such as PALB2, TP53, PTEN, and CDH1 based on family or personal history	Recommended for high-penetrance genes in high-risk patients	Recommended for high-penetrance genes in high-risk patients
VUS	VUS should not affect clinical management, but requires monitoring and potential re-evaluation	Not specified	VUS should not change the treatment, but patients should be informed about the possibility of re-evaluation
Specific populations	Not specified	Not specified	Specific guidelines for transgender and nonbinary patients with genetic predisposition
Management of non-informative results	Negative test may require further evaluation in patients with a strong family history	Not specified	Genetic counseling recommended in case of negative test but significant familial risk

ASCO, American Society of Clinical Oncology; BC, Breast Cancer; ESMO, European Society for Medical Oncology; NCCN, National Comprehensive Cancer Network; PARPi, poly (ADP-ribose) polymerase inhibitors; PV, pathogenic or likely pathogenic variant; TNBC, Triple-Negative Breast Cancer; VUS, variants of unknown significance.

If we consider only patients with a diagnosis of breast cancer at a young age, genetic counseling should be offered to all of them, regardless of tumor subtype or family history.^22^ Current clinical guidelines recommend that *BRCA* genetic testing should be offered to all individuals, regardless of gender identity, including transgender, nonbinary, and gender-diverse individuals, if they meet personal or family history criteria indicative of hereditary breast cancer, ensuring equitable access to risk assessment and tailored cancer preventive strategies. The testing results should be available before therapy initiation in all cases where the detection of a PV may influence treatment decisions, such as the consideration of risk-reducing surgeries or the use of PARPi.

The importance of knowing the presence of PVs in the *BRCA* genes in young women with breast cancer has been recently demonstrated by a large analysis within the BRCA BCY Collaboration. In this analysis, almost 5000 young women with breast cancer diagnosed at age 40 years or younger and all *BRCA* carriers were included. The main aim was to assess the benefit of knowing the presence of *BRCA* PVs before the diagnosis of breast cancer. Patients who underwent genetic testing before diagnosis had significantly smaller tumors and lower nodal involvement compared to those tested at the time of breast cancer diagnosis. The 8-year disease-free survival (DFS) and overall survival (OS) were 73.3% (95% CI 67.3–78.4) and 90.7% (95% CI 86.5–94.0) in the *BRCA* test-before-diagnosis group and 70.4% (95% CI 67.5–73.1) and 87.4% (95% CI 85.2–89.4) in the *BRCA* test-at-diagnosis group, respectively. OS results lost statistical significance after adjustment for potential confounders, including tumor stage (adjusted hazard ratio (aHR), 0.74; 95% CI 0.47–1.15), suggesting that the benefit of knowing the *BRCA* status is likely associated with the downstaging at diagnosis, thanks to the availability of effective screening measures for breast cancer.^23^ This study highlights the critical role of pre-diagnostic awareness of germline *BRCA* status, particularly in a population of individuals who, due to their young age, would not be qualified for regular screening measures.

In addition to *BRCA*, other genes are associated with hereditary breast cancer. Recent studies have identified *ATM, CHEK2*, and *PALB2* among the most frequent gene alterations after *BRCA* in women with a diagnosis of breast cancer before the age of 45 years.^16^ Although ordering multigene panel tests that include genes beyond *BRCA* is becoming more common, particularly in patients diagnosed with breast cancer at a young age, the identification of PVs in other genes can add complexity, as well as the increased risk of identifying variants of unknown significance (VUS), making risk management recommendations more challenging.^24,25^ The current guidelines underscore the importance of offering a multigene panel test to appropriately selected patients affected by breast cancer to inform them on screening and follow-up procedures, risk-reducing surgeries, and familial risk assessment. However, the guidelines also highlight major clinical challenges, including the complexities of interpreting VUS, the potential for overdiagnosis from moderate-risk gene findings, and the limited availability of genetics expertise to support pre- and post-test counseling.^19^

### Surveillance and preventive strategies

The primary objectives of risk management in individuals carrying PVs in the *BRCA* genes are multifaceted. These include reducing the likelihood of cancer development through primary preventive strategies, including risk-reducing surgeries, as well as facilitating early detection of malignancies via secondary preventive measures.^26^

**Figure 2.**
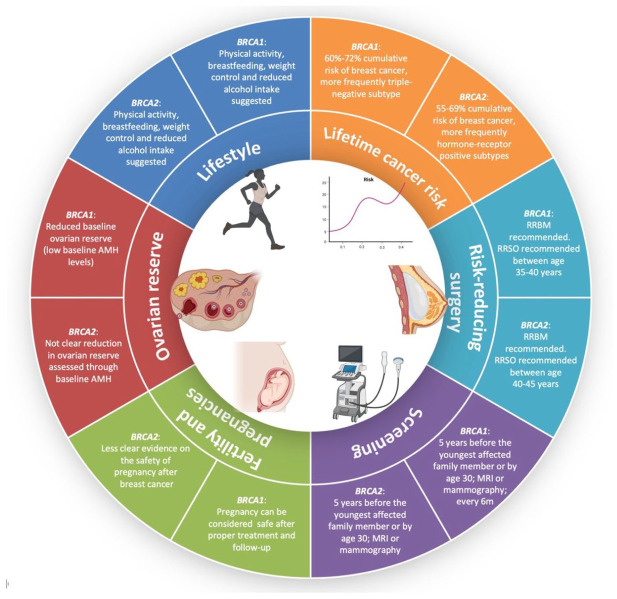
Clinical, reproductive, and lifestyle implications of carrying germline pathogenic or likely pathogenic variants in the *BRCA1* or *BRCA2* genes in young women. Source: Created in BioRender.^6^ AMH, anti-mullerian hormone; RRBM, risk-reducing bilateral mastectomy; MRI, magnetic resonance imaging; RRSO, risk-reducing salpingo-oophorectomy.

In terms of primary prevention of breast cancer, *BRCA* carriers should be informed first about lifestyle recommendations (Figure 2). In particular, physical activity and weight control should be encouraged; breastfeeding is recommended in women after a pregnancy, while limited alcohol intake should also be suggested.^26–28^ The data on the use of hormone-based contraception are mixed: while they may slightly increase the risk of breast cancer, this treatment has been shown to reduce the incidence of ovarian cancer.^26^

Risk-reducing surgeries in young healthy *BRCA* carriers are the most effective method of reducing the risk of developing breast and/or ovarian cancer (Figure 2).^29^ The advantages of undergoing a risk-reducing mastectomy are particularly relevant when performed at around 30 years of age.^30^ RRBM has been shown to reduce the lifetime risk of breast cancer by more than 90% in high-risk populations.^31^ Over time, several different approaches for RRBM have been developed to improve both aesthetic and survival outcomes, including skin-sparing and nipple-sparing techniques. Risk-reducing nipple-sparing mastectomy is particularly notable for its positive effects on patients’ psychosocial well-being, sexual health, and quality of life.^32,33^ Although several studies have investigated the potential impact of RRBM on survival, the available data remain controversial. While RRBM significantly reduces the incidence of breast cancer in high-risk individuals, no clear survival benefit has been consistently demonstrated. In individuals already diagnosed with breast cancer, RRBM has shown a dramatic reduction in contralateral cancer risk (91%–93%), but its OS benefits remain debated.^34^ In a recent analysis within the *BRCA* BCY Collaboration, 55.0% of 5290 young *BRCA* carriers with breast cancer underwent RRBM. RRBM was associated with significantly improved OS (aHR 0.65, 95% CI 0.53–0.78), along with decreased risks of DFS and breast cancer-free interval (BCFI) events. The survival benefit was consistent across *BRCA1* and *BRCA2* carriers, independent of age at BC diagnosis, tumor subtype, size, or nodal status. In addition, RRSO was associated with overall improved OS (aHR 0.58, 95% CI 0.48–0.71), particularly benefiting *BRCA1* carriers and patients with triple-negative breast cancer (TNBC).^35^ Although the decision to undergo RRBM remains a complex and highly individualized choice to be guided by an informed discussion between patients and healthcare providers considering both medical evidence and personal preferences, these recent data should be clearly disclosed taking into account the presence of two critical risk factors (i.e. young age at first breast cancer diagnosis and presence of *BRCA* PVs) for the potential development of a subsequent second primary breast cancer.

For individuals who choose to defer or decline RRBM, in addition to screening strategies, pharmacological risk reduction can be discussed. In premenopausal women, daily treatment with selective estrogen receptor modulators (SERMs) such as tamoxifen or raloxifene represents the only available option.^36–38^ These therapies have demonstrated to significantly lower the incidence of breast cancer by targeting the hormonal pathways involved in tumor development, providing a non-surgical yet impactful approach to personalized cancer prevention.^36–40^

For secondary prevention, breast cancer screening in *BRCA* carriers is suggested by all major international guidelines. In particular, ESMO guidelines suggest performing breast cancer screening with imaging evaluations that should begin 5 years before the youngest affected relative, or at the latest at age 30 years (Figure 2). Annual MRI is the imaging of choice because of its greater sensitivity compared to all other exams.^19^ Considering the tendency to the rapid development of tumors in patients carrying PVs in *BRCA* genes, bi-annual screening for *BRCA1* carriers and annual screening for *BRCA2* carriers is recommended, if possible alternating this examination with breast echography between ages 30 and 39 years or mammography over age 40 years.^19^ For the NCCN guidelines, the optimal surveillance for *BRCA* carriers under 30 years remains uncertain, with a preference for screening with MRI; between 30 and 75 years, annual mammography and MRI are indicated.^9^

## Features, prognosis, and treatment implications of breast cancer in young *BRCA* carriers

### Breast cancer characteristics and prognosis

Several preclinical and clinical data have shown that breast cancer in *BRCA* carriers appears to have different features than malignancies arising in patients with sporadic disease. Young *BRCA1* carriers are at a significantly increased risk of developing hormone receptor-negative breast cancer,^41^ while *BRCA2* carriers are more frequently diagnosed with hormone receptor-positive disease.^42^ Another study including young women with breast cancer showed that 74.4% of *BRCA1* carriers are affected by hormone receptor-negative disease as compared to 15.5% of those harboring *BRCA2* PVs.^23^

Several studies have shown that the presence of a *BRCA* PV does not appear to independently influence overall breast cancer prognosis; however, it should be highlighted that among *BRCA* carriers, clinical outcomes vary depending on the breast cancer subtype. A meta-analysis of 66 studies assessing the survival of patients with breast cancer carrying or not a *BRCA* PV showed a tendency toward a worse breast cancer-specific survival (BCSS) and OS for *BRCA* carriers. The pooled 10-year absolute BCSS difference for *BRCA1* and *BRCA2* carriers compared to wild-type patients was 6.8% (HR 1.12; 95% CI 0.71–1.53) and 4.7% (HR 1.09; 95% CI 0.54–1.65), respectively. However, the results were heterogeneous, and the evidence was judged to be uncertain.^43^ The POSH study prospectively included 2733 young patients with breast cancer diagnosed between 2000 and 2008, of whom 338 patients were *BRCA* carriers (12% of the cohort; 201 with *BRCA*1 and 137 with *BRCA*2 PVs). After a median follow-up period of 8.2 years, multivariable analyses revealed no significant differences in OS between patients harboring or not *BRCA* PVs.^7^ Another study evaluated 10-year OS in patients with early-onset breast cancer, with particular attention to *BRCA1* carriers. Out of 3345 women diagnosed with stage I–III breast cancer at age 50 or younger, 7.0% were identified as *BRCA*1 carriers. The 10-year OS for *BRCA1* carriers was 80.9%, marginally lower than the 82.2% observed in non-carriers (aHR 1.81; 95% CI 1.26–2.61; *p* = 0.002). For *BRCA*1 carriers with small, node-negative tumors, the 10-year OS reached 89.9%.^44^

While overall there seems to be no difference between the prognosis of patients harboring *BRCA* PVs and those with sporadic disease, the prognosis of young *BRCA* carriers may vary according to the tumor subtype. *BRCA* carriers with hormone receptor-negative disease tend to experience more favorable outcomes compared to those with sporadic disease, potentially due to the distinct biology and sensitivity of *BRCA*-associated tumors to DNA-damaging therapies.^45^ In contrast, young *BRCA* carriers affected by hormone receptor-positive breast cancers exhibit features of increased biological aggressiveness and a worse prognosis when compared to the sporadic counterparts.^45^ A recent large analysis by Arecco et al., including patients ⩽40 years at diagnosis of breast cancer, evaluated the role of hormone receptor expression in the prognosis of young *BRCA* carriers. In this analysis, >4500 young *BRCA* carriers from 78 centers worldwide were included, of whom 2143 (45.5%) had hormone receptor-positive and 2566 (54.5%) hormone receptor-negative disease. Patients with hormone receptor-positive disease had higher rates of distant recurrences (13.1% vs 9.6%, *p* < 0.01) but lower rates of second primary breast cancer (9.1% vs 14.7%, *p* < 0.01) compared to those with hormone receptor-negative breast cancer. The 8-year DFS was 65.8% (95% CI 63.4–68.2) for hormone receptor-positive and 63.4% (95% CI 61.2–65.6) for hormone receptor-negative breast cancer, while the 8-year OS was 88.1% (95% CI 86.3–89.7%) in patients with hormone receptor-positive and 87.1% (95% CI 85.5–88.5) in those with hormone receptor-negative disease.^46^ Looking specifically at the prognosis according to breast cancer subtypes, 612 (14.0%) patients were classified as having luminal A-like disease, 1038 (23.8%) luminal B-like disease, 2373 (54.4%) TNBC, and 340 (7.8%) HER2-positive disease.^[Bibr bibr46-17588359251368733]^ Patients affected by luminal A-like breast cancer had the worst DFS (60.8%) compared to all the other breast cancer subtypes (TNBC 63.5%, HER2-positive 65.5% and luminal B-like 69.7%).^46^ Previous smaller studies reported similar results, suggesting that breast cancer in young *BRCA* carriers exhibits different biological characteristics.^47^

Given these subtype-specific prognostic differences in young *BRCA* carriers, further research has examined how *BRCA* PVs affect survival outcomes in patients treated with neoadjuvant or adjuvant chemotherapy. One study specifically evaluated the prognostic value of *BRCA* PVs in breast cancer patients treated with chemotherapy in these settings, comparing 266 *BRCA* carriers (171 *BRCA*1 and 95 *BRCA2* carriers) with 659 noncarriers. Across the entire cohort, *BRCA* carriers demonstrated a better DFS compared to noncarriers (HR 0.63; 95% CI 0.44–0.90, and HR 0.72; 95% CI 0.47–1.1, respectively). Moreover, in patients with TNBC, *BRCA* carriers experienced significantly longer DFS (aHR 0.50; 95% CI 0.28–0.89 for *BRCA1*; and aHR 0.37; 95% CI 0.11–1.25 for *BRCA2; p* = 0.034) and disease-specific survival (aHR 0.42; 95% CI 0.21–0.82 for *BRCA1*; aHR 0.45; 95% CI 0.11–1.9 for *BRCA2; p* = 0.023). However, in the non-TNBC group, having a *BRCA* PV did not appear to significantly impact survival outcomes. These findings suggest that *BRCA* germline PVs are associated with improved survival, specifically in women diagnosed with TNBC.^48^ De Talhouet et al. reported no significant influence of *BRCA* PVs on pathological complete response (pCR) rates in HER2-positive or hormone receptor-positive/HER2-negative diseases. In particular, hormone receptor-positive tumors in *BRCA2* carriers appeared to be less responsive to chemotherapy, with an observed response rate of only 7%.^48^

### Genomic testing in BRCA carriers

Since the 2016 guidelines on breast cancer biomarkers, emerging evidence has refined the use of genomic assays, optimizing their application based on menopausal status, age, and tumor characteristics.^49^ Shah et al. compared patients with or without *BRCA* PVs who underwent Oncotype Dx testing, stratifying by age and tumor size. Their findings revealed that *BRCA* carriers had a higher Oncotype Dx recurrence score (RS) than noncarriers, with a higher proportion of high-risk tumors in the mutated group.^50^ A recent meta-analysis by Davey et al. included five studies with 4286 patients to assess the genomic differences in tumors arising in *BRCA* carriers compared to sporadic diseases. In this analysis, 7.8% of included patients were *BRCA* carriers (333/4286) and used the Oncotype Dx to calculate the RS. The mean RS in *BRCA* carriers was 25 (range 10–71) versus 18.4 in cases of sporadic disease (range 0–62). Using Oncotype Dx matching for age and tumor size, *BRCA* carriers and non-carriers affected by HR-positive node-negative breast cancer, the median RS was higher for cases versus controls (24 vs 16; *p* < 0.0001). Carriers had more high-risk diseases (28% vs 7%), intermediate-risk diseases (56% vs 36%), and fewer low-risk diseases (16% vs 57%).^51^ Kurian et al. analyzed 37,349 patients with hormone receptor-positive/HER2-negative breast cancer, linking germline test results with the 21-gene Oncotype Dx assay. Among them, 714 patients were carriers of PVs in *BRCA1*, *BRCA2*, *PALB2*, *ATM*, *CHEK2*, or Lynch Syndrome genes. *BRCA1* carriers showed the highest mean RS (26.7 for age ⩾50 years and 36.7 for age < 50 years), followed by *BRCA2* carriers (23.3 for age ⩾50 and 24.1 for age <50 years). For patients <50 years of age, 89.4% of *BRCA1* carriers had a RS ⩾ 16. *BRCA2* and *PALB2* carriers showed similar trends but with a lower mean RS than *BRCA1* carriers.^52^

A retrospective study by Yerushalmi et al. analyzed genomic differences between tumors in *BRCA* carriers versus sporadic disease in patients with hormone receptor-positive/HER2-negative early breast cancer. A total of 81 *BRCA* carriers were included and compared to a commercial database of 799,986 samples. With the 21-gene Oncotype Dx assay, the median RS in *BRCA* carriers was 25 (range 18–35), significantly higher than the median of 16 (range 11–22) observed in sporadic diseases (*p* < 0.001). Even in this case, *BRCA* carriers had a higher incidence of high-risk (49.4% vs 16.4%) and intermediate-risk (41.9% vs 47.2%) disease, while low-risk was less common (8.6% vs 36.4%). The expression of 12 out of 16 genes differed significantly between the groups, with a profile characterized by higher proliferation and invasiveness in *BRCA* carriers. The comparison between *BRCA1* and *BRCA2* PVs showed a higher median RS in *BRCA1* carriers (29 vs 24), but the differences were not statistically significant.^53^

Taken together, these results show that hormone receptor-positive breast cancer in *BRCA* carriers appears to have biological features of greater aggressiveness compared to sporadic disease. However, data on alternative genomic assays such as EndoPredict and Breast Cancer Index (BCI) in *BRCA* PVs carriers are lacking, highlighting a relevant knowledge gap in assessing endocrine sensitivity and risk of late recurrences in this subgroup.

### Treatments

#### Chemotherapy

*BRCA*-related tumors are characterized by a high sensitivity to DNA-damaging chemotherapy, especially in hormone receptor-negative tumors.^54^

In the neoadjuvant setting, several trials evaluated the role of platinum agents in combination with chemotherapy in *BRCA* carriers. Three meta-analyses assessed the benefit of platinum agents. The first meta-analysis included nine trials (*n* = 2109 patients) and examined the impact of adding a platinum agent to neoadjuvant chemotherapy in patients with TNBC. While the inclusion of a platinum agent significantly increased pCR rates in TNBC overall, in *BRCA* carriers, the addition of carboplatin showed no apparent significant benefit.^55^ Similarly, a meta-analysis of 20 studies evaluated the impact of platinum-based chemotherapy in early TNBC, including *BRCA* carriers. While platinum-based chemotherapy significantly improved DFS and OS in both the neoadjuvant and adjuvant settings, subgroup analyses showed no apparent benefit in DFS in *BRCA* carriers.^56^ Another meta-analysis that included exclusively *BRCA* carriers from 31 studies (*n* = 619) showed higher pCR rates when platinum-based chemotherapy was used in combination. Platinum-based agents combined with standard chemotherapy (anthracycline, taxane, and cyclophosphamide) achieved a pCR rate of 62% (95% CI 0.48–0.76). Similarly, the carboplatin and taxane regimen yielded a pCR rate of 63% (95% CI 0.47–0.79) compared to platinum monotherapy, which showed a lower pCR rate of 53% (95% CI 0.30–0.76).^57^ Secondary analyses from the BrighTNess trial showed that the pCR benefit of adding carboplatin to standard neoadjuvant chemotherapy in TNBC patients was independent of *BRCA* status.^58^ The INFORM phase II trial compared neoadjuvant cisplatin (CDDP) and doxorubicin-cyclophosphamide (AC) in 117 *BRCA* carriers with stage I–III HER2-negative breast cancer, of whom 82 had TNBC. pCR rates were 18% for CDDP and 26% for AC. Single-agent CDDP did not show superior efficacy over AC in *BRCA* carriers with TNBC or hormone receptor-positive/HER2-negative disease.^59^ While *BRCA* carriers are known to have heightened sensitivity to platinum-based chemotherapy, all this evidence suggests that adding a platinum agent to regimens already containing DNA-damaging chemotherapy like anthracyclines and cyclophosphamide may not offer additional benefits.

Patients with *BRCA1* PVs and hormone receptor-negative tumors seemed to have a better response to neoadjuvant chemotherapy, as evidenced by higher rates of pCR, and improved long-term survival rates.^60^ *BRCA* carriers are significantly more likely to receive adjuvant chemotherapy than individuals with sporadic breast cancer (85.1% vs 60.0%; *p* < 0.005). However, despite these treatment differences, multivariable analyses showed no significant differences in the risk of distant recurrence or mortality between *BRCA* carriers and those with sporadic disease, regardless of chemotherapy treatments.^61^ In patients with TNBC, previous studies consistently showed that *BRCA1* and/or *BRCA2* carriers exhibit higher pCR rates after neoadjuvant chemotherapy.^59,62–65^ These findings suggest that tumors associated with *BRCA* PVs are more sensitive to DNA-damaging chemotherapy, which may explain why the additional benefit of adding a platinum agent is less pronounced for these patients. In other words, the heightened sensitivity of *BRCA*-associated tumors to DNA-damaging chemotherapy may limit the advantages of adding a platinum agent to standard anthracycline- and taxane-based chemotherapy in these cases.

Notably, in young women, chemotherapy increases the risk of gonadotoxicity. In a study in mice carrying PVs in *BRCA* or other DNA repair genes, co-administration of carboplatin and paclitaxel caused significant ovarian reserve depletion, revealing potential increased fertility risks.^66^

#### Immunotherapy

Breast cancer in *BRCA* carriers is known to have an elevated expression of programmed death-ligand 1 (PD-L1), which serves as a compensatory mechanism for inhibiting T-cell activation within tumor sites.^67^ This upregulation is linked to genomic instability and the resulting expression of neoantigens on the tumor surface, leading to an increase in tumor-infiltrating immune cells. PD-L1 expression varies between *BRCA1* and *BRCA2* carriers, being higher in *BRCA*1 carriers compared to *BRCA*2 carriers.^67,68^

Pembrolizumab is now the standard therapy, in combination with chemotherapy, in most patients with early-stage TNBC based on the results of the KEYNOTE-522.^69,70^ In the KEYNOTE-522 trial, 1174 patients with previously untreated stage II or III TNBC, neoadjuvant and adjuvant pembrolizumab combined with chemotherapy demonstrated a statistically significant improvement in 60-month OS, with an estimated survival rate of 86.6% in the pembrolizumab-chemotherapy group compared to 81.7% in the placebo-chemotherapy group.^71^ However, no specific data comparing the clinical efficacy of neoadjuvant and adjuvant pembrolizumab in *BRCA* carriers versus those with sporadic disease have been reported to date. The lack of this information is particularly critical in a curative-intent setting. Further investigation is warranted to assess the potential benefit of immunotherapy in patients harboring *BRCA* PVs.^70^ Recent real-world data comparing the KEYNOTE-522 trial regimen and standard treatment suggest that *BRCA* carriers achieve similar pCR rates regardless of the treatment regimen received.^72^ The absence of a detectable difference is likely due to the high pCR rate of 75% observed in *BRCA* carriers receiving chemotherapy only without immunotherapy.

In the metastatic setting, first-line immunotherapy is approved in association with chemotherapy, and different studies evaluated its use in combination with PARPi in *BRCA* carriers affected by metastatic TNBC. Three small trials (TOPACIO, DORA, and MEDIOLA) tested the combination of an immune checkpoint inhibitor with a PARPi, demonstrating promising efficacy in *BRCA* carriers with metastatic breast cancer.^67,73,74^

#### Target therapy

Homologous recombination deficiency (HRD) causes cells to rely on alternative, error-prone DNA repair mechanisms, which can lead to the accumulation of double-strand breaks.^[Bibr bibr75-17588359251368733]^ This explains why breast cancer driven by *BRCA* PVs has enhanced sensitivity to PARPi and DNA-damaging agents.^75^ PARPi specifically targets the enzyme responsible for repairing single-strand DNA breaks, leveraging the concept of synthetic lethality in HRD cells, resulting in replication arrest and subsequent tumor cell death. Olaparib is now approved for use in *BRCA* carriers affected by early HER2-negative breast cancer.^76^ In the adjuvant setting, the phase III OlympiA trial established the benefit of olaparib in high-risk, *BRCA* carriers with HER2-negative early breast cancer.^77^ A total of 1836 patients were randomly assigned to receive 1 year of adjuvant olaparib or placebo after local treatment and neoadjuvant or adjuvant chemotherapy. At the last update of the study presented at the San Antonio Breast Cancer Symposium, olaparib showed to significantly improve 6-year invasive DFS from 70.3% to 79.6% (HR 0.65; 95% CI 0.53–0.78), distant DFS from 75.7% to 83.5% (HR 0.65; 95% CI 0.53–0.81), and OS from 83.2% to 87.5% (HR 0.72; 95% CI 0.56–0.93).^78^ OS benefit associated with adjuvant olaparib was observed in all pre-specified subgroups, including in patients who had or had not received prior platinum-based chemotherapy, those treated in either the neoadjuvant or adjuvant setting, and among individuals harboring *BRCA1* versus *BRCA2* germline PVs or being affected by triple-negative or hormone receptor-positive/HER2-negative disease, indicating a broadly applicable therapeutic benefit regardless of baseline treatment characteristics or *BRCA* variants.^78^ These findings support the relevance of *BRCA* testing also for decisions regarding systemic anticancer therapies.

Notably, a preclinical experiment in mice suggested that olaparib may play a role in gonadal toxicity by significantly depleting primordial follicle oocytes. Olaparib did not exacerbate chemotherapy-induced ovarian follicle loss, suggesting that its impact on ovarian reserve may be independent of cytotoxic treatments.^79^

In the neoadjuvant setting, a pilot study of single-agent talazoparib in *BRCA* carriers showed a reduction in tumor volume after 2 months of treatment (median decrease of 88%).^80^ A subsequent phase II trial reported a pCR rate of 45.8% in *BRCA* carriers with TNBC after 6 months of single-agent talazoparib.^81^ Several studies explored the use of currently available PARPi in combination with chemotherapy, with limited success. The phase II GeparOLA trial evaluated olaparib (200 mg/day) with paclitaxel versus carboplatin with paclitaxel, followed by epirubicin and cyclophosphamide in patients with HER2-negative, HRD tumors.^82^ Although numerically higher pCR rates and improved tolerability were observed with olaparib-paclitaxel versus carboplatin-paclitaxel, the long-term analysis failed to demonstrate a survival advantage in the overall population.^82^ However, in *BRCA* carriers, the pCR rate was significantly higher than in non-carriers (62.7% and 41.3%, respectively; *p* = 0.047), regardless of the type of treatment received.^82^ In the phase III BrighTNess trial, in patients with stage II–III, high-risk TNBC, the primary analysis did not show a higher pCR rate with the addition of veliparib to carboplatin-paclitaxel compared to chemotherapy alone (in both cases followed by doxorubicin and cyclophosphamide) in the overall population as well as in the subgroup of *BRCA* carriers.^83^ A real-world study investigated the combination of PARPi and chemotherapy in the neoadjuvant setting, failing to demonstrate a benefit in pCR rate compared to chemotherapy alone.^60^ In the advanced disease setting, olaparib and talazoparib monotherapies have received approval for the treatment of advanced breast cancer based on the data from the OlympiAD and EMBRACA trials, respectively.^84,85^

#### Cyclin-dependent kinase 4/6 inhibitors

CDK4/6 inhibitors have a crucial role in the management of hormone receptor-positive/HER2-negative breast cancer.

In the metastatic setting, palbociclib, ribociclib, and abemaciclib are approved in combination with endocrine therapy as the standard first-line treatment.^86^ Emerging data suggest that CDK4/6 inhibitors may have reduced efficacy in *BRCA* carriers with metastatic breast cancer.^87,88^ These findings are consistent with retrospective genomic analyses that identified *BRCA1* PVs among alterations associated with resistance to CDK4/6 inhibitors in hormone receptor-positive metastatic breast cancer.^89^ Moreover, *BRCA2* PVs are associated with shorter PFS in patients with breast cancer receiving CDK4/6 inhibitors and endocrine therapy.^90^ A strong association was observed between *BRCA2* PVs and pathogenic somatic RB1 alterations, which is a known driver of resistance to CDK4/6 inhibitors.^91^

In early-stage disease, abemaciclib and ribociclib are approved as adjuvant treatments for patients with hormone receptor-positive/HER2-negative breast cancer at high risk of recurrence.^92^ In this setting, there is no clear evidence of a potential lower efficacy of CDK4/6 inhibitors in *BRCA* carriers. At the ESMO Breast 2024 congress, data in *BRCA* carriers included in the monarchE trial were presented. A total of 41 (3.5%) *BRCA* carriers were identified (abemaciclib plus endocrine therapy *n* = 20; endocrine therapy alone *n* = 21): despite the limited sample size, the results suggested a benefit of adding adjuvant abemaciclib irrespective of the presence of *BRCA* PVs.^93^

These observations have sparked interest in alternative treatment sequences. Notably, new trials in the metastatic setting, such as EvoPAR-Breast01 (NCT0638075), are exploring the strategy of administering PARPi prior to CDK4/6 inhibitors. Recent data from Safonov et al.^94^ have demonstrated that biallelic inactivation of RB1, a known resistance mechanism to CDK4/6 inhibitors, can emerge under selective pressure: upfront use of PARPi may potentially delay this resistance pathway, enhancing the efficacy of subsequent endocrine-based therapies. The differential efficacy of CDK4/6 inhibitors observed between the adjuvant and metastatic settings in *BRCA* carriers remains not fully understood. While micro-metastatic disease in the adjuvant setting may retain sensitivity to CDK4/6 inhibition, metastatic disease frequently develops primary or acquired resistance mechanisms, such as loss of retinoblastoma protein, upregulation of the cyclin E-CDK2 axis, and hyperactivation of the PI3K/AKT/mTOR signaling pathway, which collectively limit the therapeutic efficacy of CDK4/6 inhibitors.^95^ Moreover, in the metastatic setting, resistance may also be driven by tumor evolution and the emergence of adaptive mechanisms, including activation of compensatory signaling pathways and epigenetic reprogramming, which diminish dependence on CDK4/6-mediated cell cycle regulation.^95^ However, these data are not specific to *BRCA* carriers, and further studies are required to elucidate the mechanisms of resistance in this subgroup.

## Survivorship

### Fertility and pregnancy-related issues in young BRCA carriers

International guidelines recommend that comprehensive oncofertility counseling before starting anticancer treatments should be provided to all patients with cancer diagnosed at reproductive age (Figure 2).^96^ *BRCA* carriers face additional challenges regarding oncofertility.^11,97^ Specifically, it was shown that *BRCA* carriers seem to have a reduced ovarian reserve due to their deficient DNA repair mechanisms. This has been shown by indirect ovarian reserve assessments, with reduced anti-mullerian hormone (AMH) levels observed in patients with breast cancer and *BRCA* carriers versus noncarriers before starting active anticancer treatments.^97^ Moreover, the role of DNA repair mechanisms in increasing the susceptibility of these patients to the gonadotoxic effects of treatment remains a topic of debate.^98–100^ The main fertility preservation strategies in young women consist of embryo/oocyte cryopreservation or ovarian tissue cryopreservation (OTC) (Figure 2).^11^ Embryo/oocyte cryopreservation is considered the standard first option to be discussed with all young *BRCA* carriers with breast cancer.^97^ This technique requires approximately 2 weeks of stimulation with exogenous gonadotropins to achieve ovarian hyperstimulation and follicular maturation.^101^ It should be offered to patients preferably under 40 years of age (as the success in older patients is very low), who are post-pubertal, and can delay active anticancer treatment by at least 2 weeks. This technique has proven to be effective and safe, including among young *BRCA* carriers.^102,103^ A retrospective analysis by Condorelli et al. among the patients recruited in the BRCA BCY collaboration examined 168 patients harboring a *BRCA* PV and having a pregnancy after a prior history of breast cancer, of whom 22 underwent assisted reproductive technologies (ART) and 146 conceived naturally. No apparent difference in terms of obstetrical outcomes or the number of DFS events was observed among patients who used ART or had a natural conception.^104^ Notably, ART allows for discussing preimplantation genetic testing in *BRCA* carriers.^105,106^ Reassuring data were observed in the more recent update of the BRCA BCY Collaboration.^107^ Among 543 young *BRCA* carriers with a pregnancy after breast cancer, 436 conceived naturally and 107 through ART, with ART involving various procedures like oocyte/embryo cryopreservation, in vitro fertilization (IVF)/intracytoplasmic sperm injection (ICSI), and oocyte donation. At a median follow-up of 9.1 years, ART was not associated with detrimental effects on DFS; pregnancies with ART had higher miscarriage rates (11.3% vs 8.8%) but fewer induced abortions (0.9% vs 8.3%) compared to spontaneous pregnancies.^107^ Another multicenter cohort study evaluating the impact of ART in breast cancer survivors included five *BRCA* carriers out of 39 patients in the ART group and one *BRCA* carrier out of 79 in the non-exposed group. This study found no significant increase in recurrence risk among those undergoing ART compared to matched controls. In the overall population, despite a higher pregnancy rate in the ART group (59% vs 26%, *p* = 0.001), the recurrence rate was lower (7.7% vs 20.5%, HR 0.46; 95% CI 0.13–1.62, *p* = 0.23).^108^ Taken together, all these findings provide reassuring data on the feasibility and safety of ART in patients with breast cancer, including *BRCA* carriers.

Cryopreservation of ovarian tissue is a different technique that consists of the surgical removal of ovarian tissue (in general, fragments of the ovarian cortex) and its cryopreservation before the start of systemic anticancer treatments. This technique has some peculiar differences from embryo/oocyte cryopreservation: it is indicated in younger patients, usually <36 years at diagnosis as the chances of success in older women are limited; it can be proposed to prepubertal patients as it does not require hormonal stimulation. For this reason, this technique can be used in the most urgent situations where it is not possible to wait for the 2-week stimulation time needed for embryo/oocyte cryopreservation before starting anticancer therapies. This technique is challenging in *BRCA* carriers (especially in *BRCA1* carriers) because of the increased risk of ovarian cancer and the indication for RRSO at the age of 35–40 years in *BRCA1* carriers and 40–45 years in *BRCA2* carriers.^109^ Hence, this strategy should not be offered in *BRCA* carriers unless there is any contraindication to embryo/oocyte cryopreservation and the patient is diagnosed several years before the recommended age of RRSO.^105^ In *BRCA* carriers, the decision to undertake OTC involves a complex risk-benefit evaluation, as the potential for fertility preservation should be carefully balanced against the increased lifetime risk of ovarian cancer and the standard recommendation for risk-reducing salpingo-oophorectomy. While OTC may offer both reproductive and endocrine advantages, concerns remain regarding the overall safety of reimplanting ovarian tissue in this population at higher risk of developing ovarian cancer. In addition to the considerations about the controversial value of OTC in the general breast cancer population,^110^ it should be highlighted that the long-term safety and effectiveness of OTC specifically in *BRCA* carriers remain largely uncharacterized.^111^ In *BRCA* carriers, tissue reimplantation should be performed into the remaining ovary to ensure optimal restoration of ovarian function and facilitate complete removal of all ovarian tissue at the time of RRSO.^111^

The administration of a gonadotropin-releasing hormone agonist (GnRHa) during chemotherapy is a standard strategy to preserve ovarian function in young patients receiving chemotherapy. Several randomized studies evaluated the efficacy of this technique in preserving ovarian function in young patients with breast cancer. A meta-analysis based on individual patient-level data showed a reduced risk of premature ovarian insufficiency and increased chances of having a future pregnancies in patients who received a GnRHa during chemotherapy as compared to those who recevied cytotoxic therapy alone.^112^ In the PROMISE-GIM6 study, 43 out of 281 recruited patients were *BRCA* carriers (five with *BRCA1* and five with *BRCA2* PVs). Among them, the incidence of chemotherapy-induced premature ovarian insufficiency was 0% (0/4) in the GnRHa arm and 33% (2/6) in the chemotherapy alone arm, suggesting a potential protective gonadal effect of GnRHa also in *BRCA* carriers.^113^

The safety of pregnancy after breast cancer has been demonstrated by several studies.^114–116^ Recently, specific data in *BRCA* carriers have become available. The first analysis of the BRCA BCY Collaboration in 2020, including 1252 patients, reported a 10-year pregnancy rate of 19% in young *BRCA* carriers with a prior history of breast cancer, with no significant differences in DFS and OS between those who conceived and those who did not.^117^ A meta-analysis of 39 studies involving 112,840 patients with breast cancer found that survivors were less likely to conceive than the general population. However, no detrimental effect of pregnancy after BC was observed in *BRCA* PV patients.^116^ A subsequent updated analysis of the BRCA BCY Collaboration reported 659 pregnancies after breast cancer diagnosis among 4732 *BRCA* carriers included in the study.^118^ The majority of pregnancies (79.2%) occurred spontaneously, despite over 90% of patients having received prior chemotherapy. Among 470 newborns with available data, the incidence of congenital abnormalities (0.4%) was comparable to that expected in the general population. A significant interaction was observed between pregnancy and *BRCA* gene status. Specifically, pregnancy was associated with improved outcomes in *BRCA1* carriers across all analyses. In contrast, *BRCA2* carriers exhibited a potential association between pregnancy and poorer disease-free survival (aHR 1.55; 95% CI 1.12–2.16).^118^

Blondeaux et al.^119^ have recently reported data on breastfeeding in young *BRCA* carriers included in the BRCA BCY Collaboration. In this analysis, 659 young *BRCA* carriers had a pregnancy after breast cancer diagnosis, and 474 delivered a child. After delivery, 23.2% of patients breastfed, 14.4% did not breastfeed, 47.5% underwent RRBM before delivery, and 15.0% had an unknown breastfeeding status.^119^ After a median follow-up of 7.0 (interquartile range 3.6–10.5) years after delivery, no difference in cumulative incidence of locoregional and/or contralateral breast cancer events between the breastfeeding and no breastfeeding groups was observed (aHR 1.08, 95% CI 0.57–2.06, *p* = 0.82). Similarly, no impact of breastfeeding on DFS (aHR 0.83, 95% CI 0.49–1.41, *p* = 0.49) nor OS (nine OS events in patients that breastfed and three in those that did not breastfeed) was observed.^119^ Regarding the possibility of having a pregnancy after hormone receptor-positive disease, the POSITIVE trial reported primary results on the safety of temporarily interrupting adjuvant endocrine therapy to attempt pregnancy; the 3-year incidence of breast cancer events and distant recurrence was comparable between patients who discontinued treatment compared to a control-matched cohort of patients.^120^ In this study, 38 *BRCA* carriers were included; although these patients exhibited a higher number of breast cancer events, this increase was not statistically significant^114^ (Figure 2).

### Quality of life after treatments

In *BRCA* carriers, RRSO showed to significantly lower the risk of gynecological cancers, including ovarian, fallopian tube, and primary peritoneal cancers, by 80%–90%, while also reducing all-cause mortality by 77%.^19^ However, RRSO has important implications for QoL. Women who undergo RRSO experiencing surgical menopause often report more frequent and severe vasomotor symptoms, sleep disturbances, fatigue, depressive symptoms, and sexual dysfunction compared to those who transition through natural menopause.^121^ While vasomotor symptoms in natural menopause typically develop gradually and resolve within 4–5 years in most cases, surgical menopause leads to an abrupt onset of symptoms, which may be more intense. These symptoms could be exacerbated by endocrine therapy in women with hormone receptor-positive disease, leading to early treatment discontinuation.^122^ Several pharmacological and non-pharmacological approaches are available to counteract the side effects associated with estrogen deprivation.^123^

## Conclusion

The management of young *BRCA* carriers with breast cancer is challenging and requires a personalized and multidisciplinary approach.

The role of genetic counseling and early surveillance remains critical. Expanding access to genetic testing allows for earlier risk assessment, enabling the timely implementation of preventive measures such as risk-reducing surgery and enhanced screening protocols.

Although *BRCA* carriers do not seem to have a different prognosis than those with sporadic tumors when optimally treated, differences depending on tumor subtype in young *BRCA* carriers were described. These differences highlight the importance of individualized treatment approaches based on tumor biology.

Platinum-based chemotherapy and PARPi have demonstrated significant efficacy in tumors with HRD. More data are needed to understand the benefit of CDK4/6 inhibitors and immunotherapy in this special patient population.

Beyond oncological treatments, the long-term impact of therapy must be carefully managed. Oncofertility counseling and quality of life considerations play a crucial role in the survivorship care of young *BRCA* carriers and should be integrated into long-term care planning.

Bridging the gap between clinical research and routine oncology practice will be critical for optimizing patient outcomes, with multidisciplinary models involving oncologists, geneticists, fertility specialists, and psycho-oncologists being increasingly essential to address both the medical and psychosocial challenges faced by young *BRCA* carriers.

## Declarations

**Ethics approval and consent to participate:** Not applicable, as this article is a review of existing literature and does not involve any studies with human participants or animals.

**Consent for publication:** Not applicable.

**Availability of data and materials:** Not applicable.
